# Novel AP39-Loaded Liposomes Sustain the Release of Hydrogen Sulphide, Enhance Blood-Brain Barrier Permeation, and Abrogate Oxidative Stress-Induced Mitochondrial Dysfunction in Brain Cells

**DOI:** 10.2147/DDDT.S507697

**Published:** 2025-03-19

**Authors:** Mohamad Anas Al Tahan, Mandeep Kaur Marwah, Mandheer Dhaliwal, Lorena Diaz Sanchez, Hala Shokr, Manjit Kaur, Shakil Ahmad, Raj K S Badhan, Irundika H K Dias, Lissette Sanchez-Aranguren

**Affiliations:** 1 Aston Medical School, Aston University, Birmingham, UK; 2 School of Biosciences, Aston University, Birmingham, UK; 3 Pharmacy Division, University of Manchester, Manchester, UK; 4 School of Health and Care, Coventry University, Coventry, UK; 5 School of Pharmacy, Aston University, Birmingham, UK

**Keywords:** liposomes, hydrogen sulphide, AP39, reactive oxygen species, mitochondrial-targeted compounds, mitochondrial dysfunction

## Abstract

**Background:**

Neurodegenerative diseases are often linked to oxidative stress (OS), which worsen neuroinflammation and cause neuronal damage. Managing OS with gasotransmitters such as hydrogen sulphide (H_2_S) is a promising therapeutic approach to protecting brain cells from oxidative damage. AP39, a mitochondria-targeted H_2_S donor, has shown neuroprotective potential by reducing OS and improving mitochondrial function. However, its clinical application is limited due to poor stability and rapid release, necessitating a drug delivery system to enhance therapeutic efficacy.

**Purpose:**

This study aimed to develop a novel AP39-loaded liposomal formulation to provide controlled H_2_S release, facilitate AP39 permeation across the blood-brain barrier (BBB), and assess functional effects in mitigating oxidative stress and preserving mitochondrial function.

**Methods:**

AP39-loaded unilamellar liposomes were prepared via ethanol injection and characterised for size, polydispersity, and zeta potential. Entrapment efficiency was determined using HPLC, while cytotoxicity was assessed in human vein endothelial (HUVEC) and neuroblastoma (SHSY5Y) cells. Liposomal permeability, AP39 release kinetics, and cellular uptake were evaluated using a microvasculature BBB model. Mitochondrial function under oxidative stress was assessed using a Seahorse XFe24 Analyzer.

**Results:**

AP39-loaded liposomes had an average size of 135.92 ± 10.05 nm, a zeta potential of 17.35 ± 3.40 mV, and an entrapment efficiency of 84.48% ± 4.7. Cytotoxicity studies showed no adverse effects after 4 h. Cellular uptake of encapsulated AP39 was significantly higher (7.13 ± 0.28 µg) than the free form (5.8 ± 0.31 µg). The BBB model demonstrated sustained AP39 release (7.28 µg/mL vs 6.44 µg/mL for free AP39). Mitochondrial assays confirmed liposomal AP39 preserved H_2_S antioxidant properties and enhanced oxygen consumption.

**Conclusion:**

Our novel liposomal formulation encapsulating AP39 improves stability, promotes sustained release, and enhances BBB permeability while preserving antioxidant effects. These findings indicate that liposomal AP39 is a suitable therapeutic approach to further investigate in the treatment of neurodegenerative diseases.

## Graphical Abstract

**Figure:**
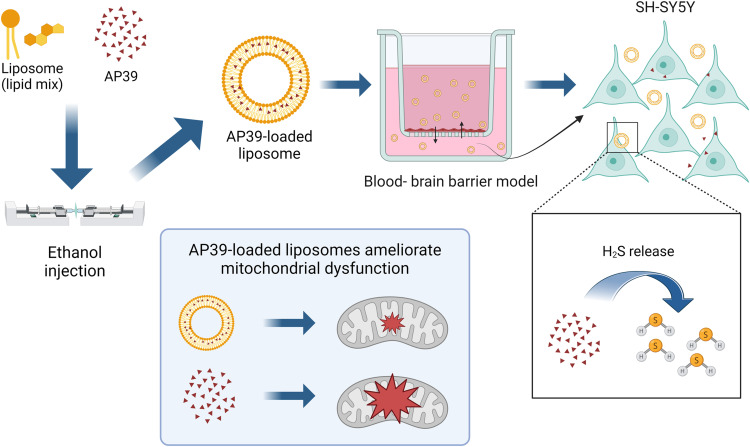

## Introduction

Neurodegenerative diseases, such as Alzheimer’s disease, Parkinson’s disease, and Huntington’s disease, are long-term conditions resulting in the progressive degeneration of neuronal cells.^1^,^2^ These conditions are incurable with treatments often limited in scope and associated range of side effects. Therefore, improving the therapeutic management for this patient cohort is of paramount importance. Neurodegenerative disorders are associated with uncontrolled inflammation that causes cell and tissue damage.^3^ Excessive Reactive Oxygen Species (ROS) production is key to the activation of a pronounced pro-inflammatory response, and mitochondria have been identified as the main players in ROS generation.^4^ This suggests that targeting the mitochondria with ROS scavengers is a promising therapeutic approach.

Hydrogen sulfide (H_2_S) is a gasotransmitter that abrogates ROS levels by quenching free radicals and increasing intracellular antioxidants.^5–7^ Previous studies have shown the potential of H_2_S-based compounds to reduce the loss of substantia nigra neurons while showing promising results in improving motor function in models of Parkinson’s disease.^8^ Increasing evidence has suggested a link between H_2_S metabolism and mitochondrial function. Studies on Alzheimer’s have shown that an imbalance in H_2_S bioavailability is accompanied by mitochondrial dysfunction.^9–13^ AP39 is a mitochondria-targeting H_2_S donor with neuroprotective effects.^14^ AP39 has been proposed as a promising drug candidate for Alzheimer’s disease, showing antioxidant properties and maintaining mitochondrial DNA integrity in murine models of disease.^9^

The translation of H_2_S donors, including AP39, into clinical practice is hindered by the practical delivery challenges associated with the gaseous nature of H_2_S, rapid H_2_S release rates, poor aqueous stability, and mitochondrial complex IV-associated toxicity when in excess.^15^ Therefore, for clinical translation, AP39 requires an adequate drug delivery system that targets the organelles of interest at a safe and sustained rate. Furthermore, the development of drug treatments for brain delivery is particularly challenging due to the presence of the blood-brain barrier (BBB). The BBB, composed capillary endothelial cells connected by tight junctions, separates the systemic circulation from the cerebral parenchyma. These intrinsic characteristics of the BBB protect the brain from undesired molecules but also reduce the permeation of drugs of interest to the brain.^16^ Formulation approaches, including the use of liposomes as nanocarriers is an emerging strategy for brain-targeted drug delivery. Previously, liposomes have been investigated for their drug delivery potential for the treatment of neurological diseases.^17^ Amongst advantages of liposomes, their structure and double lipid layer allows to incorporate and deliver large amounts of drugs, mitigate toxicity, offer a sustained release profile, and have cell targeting potential.^18^

Safe formulations for H_2_S donors that can provide sustained release of the active moiety are necessary to drive the clinical potential of H_2_S donors. Therefore, in this study, we designed AP39-loaded liposomes and investigated the feasibility of the novel carrier to provide controlled release of H_2_S, reach and accumulate in the brain, study its ability to permeate through a BBB model, and explored the therapeutic potential of this novel delivery system by investigating the ability of AP39-loaded liposomes to ameliorate mitochondrial dysfunction in an oxidative-prone environment.

## Materials and Methods

### Materials

Soy phosphatidylcholine (PC) and 1,2-distearoyl-sn-glycero-3-phosphoethanolamine-N-[amino(polyethylene glycol)-2000] (DSPE-PEG(2000)) were obtained from Avanti Polar Lipids. Cholesterol (grade ≥ 99%, catalogue #C8667), adenosine 3′,5′-cyclic monophosphate sodium salt monohydrate, 4-(3-Butoxy-4-methoxybenzyl)imidazolidin-2-one (RO 20–1724), and hydrocortisone were purchased from Sigma-Aldrich (Dorset, UK). AP39 was obtained from Cayman Chemicals (catalogue no.17100). Other reagents employed in this research; including trifluoroacetic acid, ethanol, and acetonitrile were obtained from Fisher Scientific. Ultrapure water was obtained from a Milli-Q purification system (Millipore, Billerica, MA, US). Polycarbonate filters with pore sizes of 400, 200, 100, and 50 nm were obtained from Sigma-Aldrich.

### Methods

#### AP39-Loaded Liposomes Formulation

Liposomes were prepared using the ethanol injection method described by Batzri and Korn.^19^ Briefly, egg PC, cholesterol, DSPC-PEG (16:8:X µM), and AP39 (final concentration, 5.6 µg/mL) were dissolved in ethanol. After, the organic phase obtained from the previous step was injected using a syringe pump in 1 mL of phosphate buffered saline (PBS) under magnetic stirring at temperature above 25°C (transition temperature of the lipids). Following, the lipid mixture was continuously stirred for 5 min at room temperature. The resulting mixture was extruded eight times through 400, 200, and 100 nm diameter polycarbonate membranes using an Avanti Mini Extruder, in order to produce unilamellar vesicles. The ethanol and unentrapped drug were removed by dialysis against distilled water for 24 h using Slide-A Lyzer dialysis cassettes (12–14 kDa MWCO). The mean liposome size, polydispersity index, particle charge, and deformability of liposomes were assessed using protocols established in our laboratory, as described previously.^20^

#### Determination of Liposome Entrapment Efficiency

In order to compare the entrapment efficiency of AP39 in liposomes, we determined AP39 and compared these values pre- and post-dialysis. To dissolve liposomes, acetonitrile was added at a 1:3 ratio and then, AP39 was analyzed using HPLC-UV analysis. Finally, the percentage of encapsulation efficiency for AP39, determined in liposomes was calculated using the following equation:

$$E = {{{A_d}} \over {{A_t}}} \times \mathrm{100\%}$$

*E* is the encapsulation efficiency determined in %. *A_d_* refers to the AP39 values after post-dialysis, determined in mg while A*_t_* is the total AP39 value determined before dialysis, determined in mg.

#### HPLC Methodology and Validation

To detect AP39 levels, we employed a reverse-phase HPLC, as described previously.^21^ Briefly, a Shimadzu LC-2030C Plus RoHS - Prominence-I separation module HPLC with UV detection was used. The operating wavelength used was 242 nm with a Phenomenex HyperClone™ column (5µm C18 4.6 × 150 mm column). The sample volume utilised was 2.5 μL, injected at 27°C. When stored, samples were kept at 4°C in an autosampler. For the mobile phase, we utilised a 7:1 ratio of 0.1% TFA in acetonitrile to 0.1% TFA in water, with a flow rate of 1.25 mL/min. Different stock and standard solutions of AP39 were dissolved in ethanol, with concentrations ranging from 0.0001 to 1 mg/mL. The resulting calibration curve displayed an R^2^ value of 0.998 and a linear equation of y = 5,177,240 × x – 3055 was obtained.

#### Cell Culture

SK-N-SH subclone neuroblastoma cells (SHSY5Y) (American Type Culture Collection, ATCC) at passage of 21 were cultured in RPMI 1640 containing 5% Fetal Bovine Serum (FBS), 2 mm glutamine, and 1% of Penicillin Streptomycin (Gibco, UK) cells were maintained at passage 25°C and 37°C in a 5% CO_2_ humidified atmosphere, with the medium being refreshed every 48 h.

Primary Human Umbilical Vein Endothelial (PromoCell, Cat. # C-12203) were cultured in a complete growth medium (EGM-2) (PromoCell, Cat. # C-22211) supplemented with Fetal Calf Serum 0.02 mg/mL, Epidermal Growth Factor 5ng/mL, Basic Fibroblast Growth Factor 10 ng/mL, Insulin-like Growth Factor 20 ng/mL, Vascular Endothelial Growth Factor 0.5ng/mL, Ascorbic Acid 1μg/mL, Heparin 22.5 μg/mL, Hydrocortisone 0.2 μg/mL (supplement kit, Promocell, Cat. # C-39211), and 5 mL of (10,000 U/mL) Penicillin/Streptomycin (Lonza, Cat. # LZDE17-602E). Cells were maintained at 37°C in a 5% CO_2_ humidified atmosphere. Cells were sub-cultured at 70–80% confluency and used for experiments up to passage five. Before each treatment, treatments were diluted in serum-starvation medium (M199 containing 1% FBS, Lonza, Cat. # LZBE12-119F).

#### Cell Viability

The cytotoxicity of AP39 and AP39-loaded liposomes was determined using a commercial LDH assay kit (LDH Cytotoxicity Kit II, Promocell, Germany) to measure cell viability to both HUVEC and SHSY5Y cells, subsequently to exposure to AP39 and AP39-loaded liposomes during 24 h. Cells were seeded at a density of 2 × 10^4^ cells/well into a 96-well plate and incubated overnight (37 °C, 5% CO_2_) to attach. Thereafter, media were removed and fresh media containing AP39- and AP39-loaded liposomes added for 24 h (37°C, 5% CO_2_). Following, the cell culture supernatant was removed and stored at −20°C for subsequent LDH assays. Control (vehicle) was used. To determine LDH levels, 10 μL of the collected cell supernatant were combined with 100 μL of the LDH reaction mixture (WST-1 reagent and water) for up to 30 minutes as per manufacturer’s instructions. Next, the absorbance of the reaction was read at 450 nm using a Spark^®^ plate reader (TECAN, Switzerland). These experiments were performed three independent times and results were expressed as percentage of viability, determined as follows:

Percentage of viability = cytotoxicity of control (100%) – cytotoxicity of sample.

#### H_2_S Release From Liposomal Formulation and Non-Formulated AP39

H_2_S is a potent reducing agent, able to react dyes, including the tetrazolium dye 3-(4,5-dimethyl-2-thiazolyl)-2,5-diphenyl-2H-tetrazolium bromide (MTT; Sigma, St. Louis, MO) leading to a purple-colored formazan.^22^ Using this knowledge and to evaluate H_2_S released from AP39, SHSY5Y cells were seeded at a density of 2 × 10^4^ cells/well in a 96-well plate and let attach overnight at 37°C and 5% CO_2_. Thereafter, the cells were exposed to the AP39 liposomal formulations or vehicle (final concentration of 0.5 µM).^23^ Following the set exposure times, a volume of 100 μL of cell culture supernatant was collected and combined with 100 μL of MTT (5 mg/mL), the reaction occurred for 3 h in a humidified incubator at 37°C under 5% CO_2_ atmosphere. Next, changes in absorbance were measured for up to 6 h using a Spark^®^ plate reader (TECAN, Switzerland). A two-fold serial dilution standard curve using sodium sulphide (Na_2_S) as a standard was created. Absorbance at 570 nm corresponding to H_2_S readings, were determined as change in absorbance in a 60-minute time slot and then compared normalised to the standard curve values.

#### Microvascular Endothelial Model

HUVEC were seeded (5 × 10^4^) onto 24-well polycarbonate inserts (0.4 µm pore size)^24^ and cultured for up to 5 days. Tight-junction formation was enhanced at the fifth day of cell culture by the addition of cAMP (250 µM), 17.5 µM RO 20–1724, and 550 nM hydrocortisone in the absence of serum for 24-hours prior to the initiation of the assay.^25^ Barrier integrity and formation were assessed by determining the transendothelial electrical resistance (TEER), which was measured on days 3 and 4 using a chopstick electrode (World Precision Instruments STX4, Sarasota, Florida, United States).

#### Drug Transport Assay

HUVEC were grown on 0.4 µm pore permeable inserts, as defined in section “Microvascular endothelial model” and placed into a 24-well cell culture plate. Non-formulated or liposomal AP39 was prepared at a final concentration of 5.6 µg/mL (as described in section “AP39-loaded liposome formulation”). The formulation was diluted to 1 in 10 and 200µL added to the apical (for apical-basolateral flux) or basolateral (for basolateral-apical flux) compartments, with sampling taking place from the opposite compartment. Samples were collected at intervals of 30 min for up to 1.5 h. Every time, Equal amounts of serum-free culture medium were added to maintain a consistent volume.

The AP39 concentrations were analyzed using the HPLC method described above. Additionally, total AP39 (entrapped in liposomes and free AP39) was measured following the addition of acetonitrile to the sample (1:4 acetonitrile to sample) to break down the liposomes and release the entrapped drug.

The apparent permeability (P_app_) was calculated using the equation below,

P_app_ = (dQ/dt) / C_0_×A

where dQ/dt is the amount of drug permeated per unit time calculated from the regression line of the sampling time points, C_0_ is the initial drug concentration in the donor compartment, and A (cm^2^) is the insert surface area (0.33 cm^2^).

#### Mitochondrial Oxygen Consumption

Mitochondrial function parameters were assessed using an XFe24 extracellular flux analyzer (Seahorse Biosciences/Agilent Technologies, UK) following protocols established in our lab.^14^,^21^,^26^ Briefly, SHSY5Y cells were plated at 5 × 10^4^ cells/well in V7 24 well plates (Agilent Technologies, UK) and cells left to attach overnight at 37°C in a humidified atmosphere of 5% CO_2_. Following, cells were washed, and media replaced with non-buffered DMEM (10 mm glucose, 1 mm pyruvate and 2 mm L-glutamine) to allow temperature and pH equilibrium. The addition of AP39 and/or H_2_O_2_ was performed using the available ports of the XFe24 Flux Analyzer prior to the injection of drugs or inhibitors used to calculate mitochondrial function parameters. Oxygen consumption rates (OCR) were measured after AP39/ H_2_O_2_ injection (first injection), followed by sequential injections of oligomycin (1 μM) (Sigma Aldrich, USA), carbonyl cyanide 4-(trifluoromethoxy) phenylhydrazone (FCCP) (1 μM) (Sigma Aldrich, USA), and a mixture of rotenone and antimycin A (Rot/AA) (0.5 μM) (Sigma Aldrich, USA) to inhibit ATP synthase, uncouple oxidative phosphorylation, and estimate non-mitochondrial respiration by inhibiting complexes I and III, respectively (75 μL per injection). As previously described,^27^ injections of these reagents allowed the parameters of mitochondrial function, including basal and maximal respiration, spare respiratory capacity, ATP-linked OCR, and proton leak to be determined. Data are expressed as the rate of oxygen consumption (pmolO_2_/min/μg protein) expressed as time. The concentration of proteins per well was assessed using the bicinchoninic acid (BCA) protein assay (Bio-Rad) after completion of the Seahorse assays.

#### Sample Size and Statistical Analysis

Unless otherwise stated, all results are presented as the mean ± standard deviation (SD). Sample size included at least five independent replicates (n = 5). The sample size analysed per study is stated in each figure legend and each group had an equal size. Distribution of the data was first confirm using the Shapiro–Wilk test. Following, a *t*-test or one-way analysis of variance (ANOVA) was used to determine statistically significant differences between the means tested (p ≤ 0.05) when analysing two or more groups, respectively. In the case of ANOVA, post-hoc Tukey’s multiple comparison test was applied to assess the differences between groups. All calculations were performed using the GraphPad 10.2.0 (GraphPad Inc., La Jolla, CA, USA).

## Results

### Development and Evaluation of AP39 Liposome Formulations

The therapeutic potential of H_2_S donors is hindered by their inherent biochemical characteristics; including insolubility in aqueous solutions and their rapid release rate of the active gaseous compounds. To address this issue, liposomes loaded with AP39 have been formulated to provide a novel solution. Our first approach was to assess the physicochemical properties of AP39 and guide the development of liposomal formulations. Our results showed the apparent partition coefficients for AP39 in water and PBS at pH 7.4 to be 0.34 ± 0.08 mg/mL and 0.15 ± 0.03 mg/mL, respectively (Table 1). We also determined other physicochemical parameters, including log P. A negative log P value indicates a higher affinity for the aqueous phase, whereas a positive value denotes a higher affinity for the lipid phase. AP39, was observed as a hydrophobic drug, with low solubility in both, water and PBS (pH 7.4; Table 1).

**Table 1:** Physicochemical Properties of AP39. Results are Presented as the Mean ± Standard Deviation. n=4 Independent Batches

Media	Parameter	Value
Water at 37°C	Solubility × 10^3^ mg/mL	0.34 ± 0.08
	K_o/w_	400.27 ± 35.06
	LogP	2.60 ± 0.04
PBS at 37°C	Solubility × 10^3^ mg/mL	0.15 ± 0.03
	K_o/w_	630.20 ± 53.95
	LogP	2.80 ± 0.04

The physicochemical characteristics of liposomes, including their diameter, polydispersity, zeta potential, and drug entrapment efficiency, are important for drug release, permeation, and efficacy. Our study focused on these characteristics and determined the impact of the inclusion of AP39 on liposomal characteristics. The presence of AP39 in the liposome bilayer was not observed to significantly affect the diameter of the resulting liposomes, polydispersity, or charge, determined by the zeta potential (Table 2). The entrapment efficiency of AP39 within the liposome was 84.48 ± 4.7%.

**Table 2:** Liposome Characteristics Showcasing Size, Polydispersity, Zeta Potential, and Entrapment Efficiency. Data Represents Mean ± SD. n=6 Independent Batches

Characteristic	Empty Liposomes	AP39 Loaded Liposomes
Size (nm)	130.74 ± 8.95	135.92 ± 10.05
Polydispersity index	0.24 ± 0.04	0.25 ± 0.01
Zeta potential (mV)	18.86 ± 2.05	17.35 ± 3.40
Entrapment efficiency (%)	n/a	84.48 ± 4.7

### Cell Toxicity

**Figure 1:**
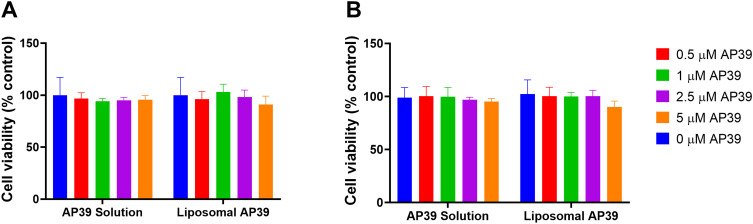
Cell viability evaluated on (**A**) HUVEC and (**B**) SHSY5Y cells following the application of AP39 in solution (non-formulated) and formulated AP39 into liposomes, assessed by LDH release. The percentage release of LDH (cytotoxicity) of each sample was subtracted from 100% viability of control (after 4 h of exposure). Results are expressed as mean ± SD and analysed by one-way ANOVA. n = 5 independent batches.

Non-formulated AP39 and AP39-loaded liposomes were applied to HUVEC and SHSY5Y to assess their impact on cells’ viability after 4 h of exposure. Cell viability was measured using the LDH assay. No statistically significant difference was observed in cell viability (p ≥ 0.05) across the concentration range of 0.5–5 µM for both, non-formulated and liposomal AP39, which suggest that no cytotoxic effects result from the liposomes and/or AP39 (Figure 1).

### Cellular Uptake

**Figure 2:**
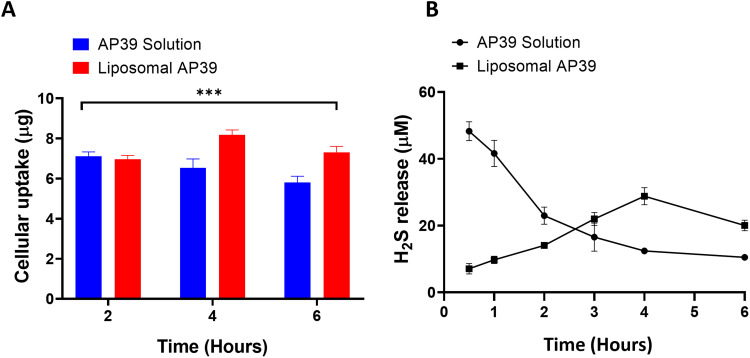
Liposomal AP39 provided a slow and sustained (**A**) cellular uptake of AP39 and (**B**) release of H_2_S from formulated AP39 compared to non-formulated AP39 on SHSY5Y cells. Hourly H_2_S release values are plotted with curve-fitting results to highlight the donor compound decomposition. Results are expressed as mean ± SD and analysed by one-way ANOVA. n = 5 independent batches. ***p ≤ 0.001.

The cellular uptake of AP39 was evaluated in SHSY5Y using both AP39 in solution and AP39-loaded liposome at different time points (Figure 2A). The maximum cellular uptake for the liposomal AP39 was observed at 6 hours (7.13 ± 0.28 µg) which was significantly higher (p = 0.0001) compared to AP39 in solution (5.8 ± 0.31 µg).

### H_2_S Uptake and Release From Liposomal Formulation and Non-Formulated AP39

To investigate whether liposomal AP39 had the potential to control the release of the active gasotransmitter, H_2_S, SHSY5Y cells were incubated with either liposomal AP39 or non-formulated AP39 in solution, with a final concentration of 0.5 µM and the levels of H_2_S were measured over 6 h. The maximum release of H_2_S was determined every 30 min (Figure 2B). Non-formulated AP39 released H_2_S rapidly with a peak release at 30 min. In contrast, AP39-loaded liposomes showed peak release at 4 h. Moreover, non-encapsulated AP39 showed a H_2_S release of 48.27 ± 2.83 µM at 30 min, which was significantly higher than that of the encapsulated AP39, with H_2_S release of 28.79 ± 2.54 µM (p = 0.001). These results show that the maximum release of H_2_S occurs rapidly in non-formulated AP39, as opposed to its formulated counterpart, suggesting the controlled release properties of the liposome formulation.

### Microvascular Endothelial Model and AP39 Permeability Assay

**Figure 3:**
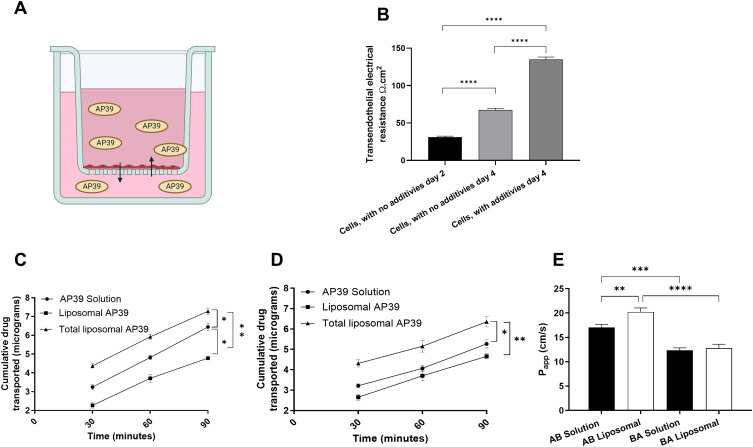
Liposomal AP39 formulation enhances the directional permeability of AP39. (**A**) AP39 permeation across a microvascular endothelial barrier model mimicking the Blood-Brain Barrier (**B**) TEER values measured following growth of HUVEC on permeable cell culture inserts (24-well, 0.33 cm2) under static conditions, before and after tight junction inducers. (**C**) AP39 apical to basolateral transport. (**D**) basolateral to apical transport. (**E**) Associated apparent membrane permeability (P_app_) values in both directions. Results are expressed as mean ± SD and analysed by one-way ANOVA. n = 6. *p ≤ 0.05, **p ≤ 0.01, ***p ≤ 0.001, and ****p ≤ 0.0001.

Prior to our in vitro studies on AP39 permeability across the BBB in vitro (Figure 3A), we confirmed the formation of a high-resistance microvascular endothelial barrier by assessing the TEER values displayed in our BBB model. This first step allowed for the generation of a consistent BBB in vitro (Figure 3B). On day 4, TEER values significantly increased from to 67.47 Ω.cm^2^ ± 2.25 to 135.1 Ω.cm^2^ ± 3.09 Ω.cm^2^ (p = 0.0001) (Figure 3B).

Permeability of AP39, of both, non-formulated and formulated into liposomes, showed AP39 liposomal formulation significantly increased the total cumulative amount of AP39 transported/permeated in the AB direction when compared to the non-formulated AP39 (Figure 3C), 7.28 ± 0.17 compared with 6.44 ± 0.19 µg/ mL over 90 min (p = 0.05). Moreover, liposomes were able to control the release of H_2_S. After 90 minutes, 4.78 ± 0.09 mg/mL free AP39 was detected. A similar trend was observed in the BA direction, with liposomes increasing the total amount of drug permeated after 90 min, 6.06 ± 0.25 compared with 5.27 ± 0.19 µg/ mL (p = 0.05) (Figure 3D). Moreover, liposomes were able to control the release of AP39, only 4.65 ± 0.13 µg/ mL of free AP39 was detected after 90 min. The apparent membrane permeability (P_app_) of the non-formulated AP39 was lower in both the AB and BA directions. Specifically, the apparent permeability (P_app_) for the non-formulated AP39 determined was P_app,_ AB: 17.03 ± 0.61 × 10^−6^ cm/s and P_app_ BA: 12.35 ± 0.50 × 10^−6^ cm/s while the liposomal AP39 formulated was P_app_ AB: 20.22 ± 0.82 × 10^−7^ cm/s and P_app_ BA: 12.78 ± 0.81 × 10^−7^ cm/s. This results in an influx ratio of 1.38 and 1.58 for the non-formulated AP39 and liposomal AP39, respectively (Figure 3E) suggesting that liposomal formulation enhances the directional permeability of AP39, indicating improved transport properties.

### Mitochondrial Oxygen Consumption

**Figure 4:**
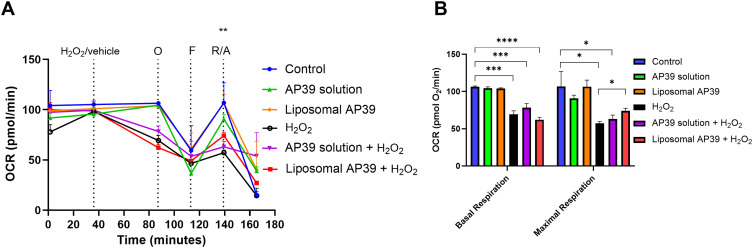
Mitochondrial oxygen consumption rates determined in SHSY5Y exposed to AP39 (in formulation or not) and in combination with H_2_O_2_ (300 µM) for 1h. (**A**) Sequential injections of Oligomycin, FCCP and mixture of Rotenone and Antimycin A allowed to calculate the parameters of mitochondrial function, (**B**) basal respiration and maximal respiration. Results are expressed as mean ± SD and analysed by one-way ANOVA. n = 5 independent batches. *p ≤ 0.05, **p ≤ 0.01, ***p ≤ 0.001, and ****p ≤ 0.0001.

Following, we confirmed whether AP39 delivered in liposomes would be able to protect against H_2_O_2_-induced mitochondrial dysfunction, retaining similar effects observed using non-formulated AP39.^14^ SHSY5Y were exposed to 300 μM H_2_O_2_ for 1h, to induce an oxidative stress prone environment, leading to a reduction in OCR levels as we previously reported.^14^ Following, SHSY5Y were exposed to either non-formulated or liposomal AP39 to evaluate their protective effects on mitochondrial bioenergetics (Figure 4A). To achieve this, we injected H_2_O_2_ alone or H_2_O_2_ in combination with either non-formulated or liposomal AP39, using the first injection port available in the XFe24 Seahorse Analyzer. This approach provides real-time evidence of the effects of our treatments on OCR. H_2_O_2_ alone reduced the baseline OCR levels (p = 0.05, compared to control and AP39), resulting in reduced levels of basal respiration (p < 0.005 vs AP39 only) (Figure 4B) and maximal respiration (p < 0.05, vs control and AP39 only). However, although co-exposure to H_2_O_2_ and AP39 resulted in reduced basal respiration, it was sufficient to significantly improve maximal respiration in the presence of liposomal AP39 (Figure 4B). These observations are consistent with previous studies showing the neuroprotective effects of A39-derived H_2_S against oxidative stress^14^ and confirm the specific role of AP39 in protecting against mitochondrial dysfunction.^9^,^14^,^28^

## Discussion

Oxidative stress is a crucial molecular mechanism implicated in the pathophysiology of neurodegenerative disorders,^29^,^30^ including Parkinson’s disease,^31^ and Alzheimer’s disease.^32^,^33^ Hydrogen sulfide (H_2_S), a gasotransmitter used in cardiovascular science, regulates ROS production by quenching free radicals.^5^,^34^,^35^ Studies have shown that treatment with H_2_S donors reduces the loss of substantia nigra neurons and slows down the development of motor dysfunction in Parkinson’s disease models.^34^ Nonetheless, H_2_S donors display practical delivery challenges associated with intrinsic biochemical characteristics of these donors, including their rapid H_2_S release rates, gaseous nature of H_2_S, poor aqueous stability, and potential toxicity when present in excess. Furthermore, the development of strategies to deliver drugs to the central nervous system is of high importance, because many drug candidates are unable to permeate the BBB. Therefore, liposomes may be useful for clinical translation of H_2_S donors. In this study, we explored the potential of formulating AP39, a novel mitochondrial-targeted H_2_S donor, into liposomes, to successfully control the release of H_2_S and protect against ROS, in SHSY5Y exposed to AP39 that permeated the BBB in the presence of pro-oxidant environment established by H_2_O_2_. Our results suggest that liposomes are a candidate for controlling the release of H_2_S and improving the transport of AP39 across the BBB, allowing the maintenance of protective effects on mitochondrial bioenergetics.

Liposomes are well-established nanosized carriers with potential for effective neuronal drug delivery.^18^ In the pipeline for clinical translation, it is imperative to ensure reproducible drug delivery rates. In this regard, several liposome characteristics including size distribution, polydispersity, charge, and drug entrapment efficiency are critical in determining their potential application in drug delivery.^36^ As previously established, a homogenous liposome preparation is crucial for an effective drug release kinetics and successful degree of tissue distribution in vivo.^37^,^38^ Furthermore, a neutral liposomal surface charge can reduce clearance^39^ however, a slight charge, as observed in the AP39 loaded liposomes in this study, is useful for avoiding particle flocculation due to electrostatic repulsion between liposomes during storage.^40^ Liposomes are controlled delivery systems.^41^,^42^ Liposomes successfully entrapped AP39 and controlled the release of H_2_S from AP39. This observation is crucial as H_2_S treatment alone can be toxic due to its fast release and high concentrations, which can be fatal.^43^ In a similar study, liposomal nanoparticles were used for the encapsulation and controlled release of the H_2_S donor ZYZ-802. Encapsulation stabilized ZYZ-802 and prolonged the release of the compounds from 30 to 36 h in vitro settin*g*. Furthermore, liposomal formulations generated significantly more H_2_S than free ZYZ-802 in all tissues over 24 h.^44^ Additionally, it should be noted that the polydispersity and zeta potential remained unchanged before and after AP39 loading due to its lipophilic and positively charged nature, which allows it to integrate into the lipid bilayer without significantly altering the surface properties of the liposomes. Since PDI reflects size distribution, AP39’s incorporation does not disrupt liposomal structure or cause aggregation, maintaining uniformity. Similarly, zeta potential is primarily influenced by lipid composition, and as AP39 localises within the bilayer rather than on the surface, it does not significantly impact surface charge.

Mitochondrial dysfunction is regarded as a crucial mechanism implicated in the onset of neurological disorders.^45^ As described above, AP39 has shown the potential to protect mitochondrial function and mitochondrial ROS generation by the targeted delivery of H_2_S. AP39 has been shown to reduce H_2_O_2_-induced mitochondrial impairment by improving mitochondrial function parameters and abrogating the generation of mitochondrial ROS.^14^ Liposomes were observed to improve mitochondrial function more than the non-formulated AP39 after 90 min. This may be due to the increased cellular uptake of AP39 as well as the controlled release nature of the formulation, which enhances the bioavailability and stability of AP39, thereby ensuring a more sustained and targeted therapeutic effect.^23^ Consequently, liposomal formulation of AP39 holds promise for more efficient protection against mitochondrial dysfunction in neurological disorders, which remains to be investigated in vivo.

The development of drug delivery systems for brain-specific application is particularly challenging because of the presence of the BBB. Liposomes have been used as delivery vehicles to overcome BBB.^46^,^47^ Liposomes offer advantageous features including the ability to incorporate and deliver large amounts of drug, mitigate toxicity, reduce drug breakdown, offer a sustained release profile, and have targeting potential.^18^ Liposomes were observed to increase the rate of AP39 permeation across a cellular barrier model; however, they provided controlled release of AP39 from the liposome, as defined by cumulative drug detection and an increase in P_app_. A similar study, in which nerve growth factor (NGF) was encapsulated into liposomes to protect it from enzyme degradation in vivo was observed to promote its permeability across the BBB.^46^ These liposomes also contained RMP-7, a ligand for the B2 receptor, on brain microvascular endothelial cells, which may have aided permeation. In this study, lower overall efflux values were observed. This may be due to drug sinkage and could be explored further with the incorporation of a shaker plate within the incubator. The targeting potential of liposomes in this study is yet to be explored, and the positive data generated suggests that this could further aid drug delivery in the treatment of neurological disorders. Furthermore, studies exploring the controlled release potential of liposomal AP39 in vivo would be useful to ascertain the release profiles of both AP39 and H_2_S.

## Conclusion

In this study, we developed unilamellar liposomes to facilitate the delivery of AP39, a hydrogen sulfide (H_2_S) donor, across the blood-brain barrier, targeting its neuroprotective potential against mitochondrial dysfunction. Our results indicated that AP39-loaded liposomes did not negatively affect cell viability over a four-hour exposure period. Importantly, the cellular uptake of the loaded liposomes was significantly greater (7.13 ± 0.28 µg) compared to non-loaded AP39 (5.8 ± 0.31 µg). The liposomes exhibited a controlled and sustained release of H_2_S, with a cumulative release of 7.28 µg/mL for the loaded form versus 6.44 µg/mL for the free compound. Additionally, AP39 encapsulation improved maximal respiration, offering enhanced protection against hydrogen peroxide-induced mitochondrial dysfunction compared to the non-encapsulated form. These findings confirm that AP39-loaded liposomes can effectively regulate H_2_S release while bolstering mitochondrial bioenergetics. Moreover, the preservation of AP39’s neuroprotective effects within the liposomal formulation underscores the translational potential of this delivery system. Future investigations should focus on the clinical implications of these liposomes, particularly their long-term effects on neuronal health and ability to alleviate oxidative stress in neurodegenerative models. This innovative approach may pave the way for new therapeutic strategies to improve patient outcomes in conditions linked to ROS-induced neuronal damage.

## Data Availability

All the data involved in this study are available in this published article.

## Abbreviations

BBB, blood-brain barrier; H_2_S, hydrogen sulfide; ROS, reactive oxygen species; HUVEC, human umbilical vein endothelial cells; PBS, phosphate-buffered saline; TEER, transendothelial electrical resistance; Rot/AA, rotenone and antimycin A; PC, soy phosphatidylcholine; DSPE-PEG(2000), 1,2-distearoyl-sn-glycero-3-phosphoethanolamine-N-[amino(polyethylene glycol)-2000].
